# Macromolecular crystallography for mammalian body temperature in support of molecular biophysics methods

**DOI:** 10.1007/s12551-025-01328-4

**Published:** 2025-08-01

**Authors:** Alice Brink, John R. Helliwell, Francois J.F. Jacobs

**Affiliations:** 1 Department of Chemistry, University of the Free State, Nelson Mandela Drive, Bloemfontein, South Africa; 2 Department of Chemistry, University of Manchester, Oxford Road, Manchester, UK

**Keywords:** Cryogenic / mammalian body temperature, Biophysical methods, Structure function, Crystallography, Dynamics, Kinetics, wwPDB data analysis

## Abstract

This review describes various molecular biophysics methods (crystallography, mass spectrometry, NMR spectroscopy, electron cryo-microscopy (cryoEM), free electron laser and X-ray photon correlation spectroscopy) which can be used to investigate the molecular structure of proteins under diverse conditions (visually summarised in the thumbnail image for the journal contents pages). We focus particularly on those which permit for the incubation and/or experimental sample analysis at mammalian body temperature (37 °C) or at physiological conditions for yet higher temperatures such as thermophiles. Crystallography, a leading method in structure elucidation, in recent decades has been dominated by structures analysed at cryogenic temperatures to ensure best resolution and crystal stability under X-ray irradiation. However, it raises the question—is the atomic structure elucidated by cryo-crystal structures truly representative of processes occurring at body temperature? This is surely an important requirement for protein–ligand binding investigations for drug discovery as protein binding may vary with temperature and indicate key aspects that could be overlooked. A review of wwPDB submissions versus sample temperature analysis clearly indicates a marked lack of atomic data obtained at 37 °C. This is not to say that 100 K cryo-crystallography ought to be replaced, in such structure-based drug discovery which is highly efficiently implemented at many macromolecular crystallographic beamlines worldwide, but favoured ligand binding events in particular for lead compounds from those surveys may provide additional valued data when studied at 37 °C.

**Graphical abstract:**

Thumbnail contents image: Molecular biophysics methods suitable for the analysis of macromolecules at body temperatures or higher

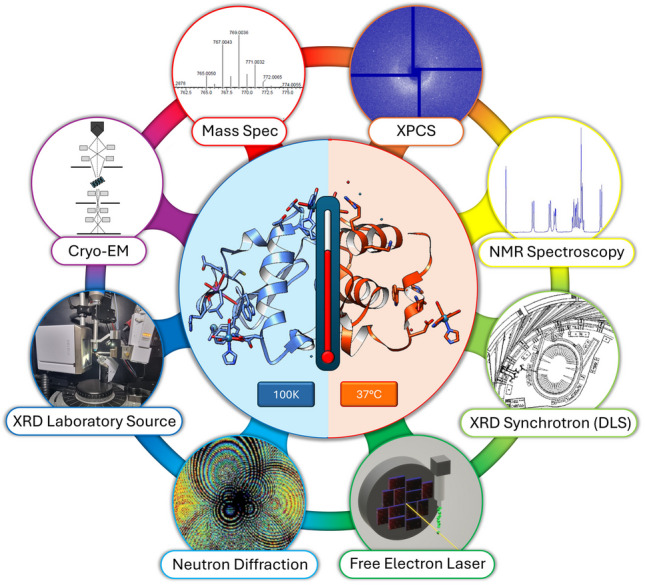

**Supplementary Information:**

The online version contains supplementary material available at 10.1007/s12551-025-01328-4.

## Introduction

### Macromolecular crystallography

In the molecular investigation of biological structures, research habits are inadvertently created in a drive to obtain the best resolution data. The natural assumption is that such data will provide the most valuable information applicable to life. This is rightly being questioned now in the field of crystallography. Are the low temperature structure analyses strictly relevant to the biology of living organisms? Crystallographic results obtained for macromolecular structures are predominantly based on X-ray diffraction data analysed at cryogenic temperatures (typically 100 K or − 173 °C) obviously significantly lower than normal physiological ranges. It is assumed that the flash-cooling process is rapid enough to kinetically trap the macromolecule and associated solvents in a room temperature equilibrium state. The relevance of assuming direct correlation of the cooled state to living biological systems has been raised by Halle, who stated: ‘*In biomolecular cryocrystallography: structural changes occur during flash-cooling: conformational switching of solvent-exposed side chains and weak ligand binding are likely artefacts…. Also, the sites for molecular recognition, ligand binding or chemical catalysis, usually involve the solvent interfacial region where consequent cryo-artefacts are expected to be most pronounced*’ (Halle 2004). Halle’s analysis indicated that many degrees of freedom are suppressed at temperatures around 200 K, meaning that the global backbone fold of the protein structure determined at 100 K may be the same at room temperature; however, subtle differences besides the fold itself may occur. Halle (2004) cited direct comparisons that had been made between low-to-room temperature diffraction data to validate regions of weak ligand binding, salt bridges, conformations of exposed side chains or solvent coupled processes (e.g. including our own study (Deacon et al. 1997)). Most recently, Guerrero et al. (2024) noted the limited number of proteins studied at high temperature and also considered the distinct effects which high pressure and high temperature had on the tyrosine phosphatase (PTP) enzyme STEP (PTPN5) with regard to effects on protein volume, patterns of ordered solvent, local backbone and side-chain conformations. Whilst changes within a protein may appear small due to these deviations from STP (standard temperature and pressure), it is important to note the complexity of a protein molecule and sub-angstrom shifts may significantly affect the protein function (Barstow et al. 2008). For example, the Ras protein has been investigated at high pressure and room temperature analysis permitting the inducement of an excited state whereby an inhibitor could bind under high pressure which was not observed at ambient (Girard et al. 2024). Additional stable conformers were also observed under high pressure (Girard et al. 2022). Such subtle structural changes need to be assessed for their significance (Kumar et al. 2015) and likewise changes of atomic displacement parameters, i.e., B factors (Helliwell 2023).

The evaluation of multiple temperature crystal structures was revolutionarily conducted on ribonuclease studied at nine temperatures from 98 up to 320 K (PDB codes 1RAT to 9RAT) (Tilton et al. 1992). These were undertaken to scrutinise protein structure differences with temperature. Unfortunately, the coordinates of the bound waters and the structure factors are unavailable. Another example of crystallographic structure analysed at various temperatures is the study on the concanavalin A protein mentioned above (Deacon et al. (1997)—within that article’s Sect. 3.9) which noted several differences between the cryo- and room temperature structures and made clear which differences in structure are due to resolution (e.g. number of bound waters increases) and those that arise from atom movements. Several of the amino-acid side chains of the concanavalin A protein had adopted largely different conformations. Most of the changes were in the poorly determined residues in the room temperature structure, especially in the flexible loop regions. It was also noted that the number of detected solvent sites had more than doubled, to 319 at 110 K as compared with 149 at 293 K (Deacon et al. 1997). There were also 20 non-matching waters in the room temperature structure which were connected to the movement of side chains by freezing out of the low-energy conformations of Asp82, Ser117, His121 (in a loop region), Lys135 and Thr196. In this case, there were no major differences in either the concanavalin A protein or solvent structure around the saccharide-binding sites.

On the other hand, utilising a high-throughput fragment screening approach, a large number of small-molecule fragments (143 ligands which were soaked at ambient temperature) bound to protein tyrosine phosphatase, PTP1B was analysed at room temperature and showed significant differences such as fewer fragments bound, lower occupancy, different binding poses and changes in solvation, in comparison to the cryo-obtained data (Skaist Mehlman et al. 2023).

In terms of the novelty for undertaking higher temperature protein X-ray crystallography, we mention that within an undergraduate physics project at York University in the mid-1980s, we showed that X-ray diffraction data could be recorded from a crystal of phenol insulin (Derewenda et al. 1989) up to 50 °C. Also, that at about 55 °C the diffraction pattern disappeared but which returned on cooling.

Multiple temperature structural studies of proteins have also been done previously for studying aspects of protein folding and unfolding (Scalley and Baker 1997; Feller 2018; Thompson 2023). There is also the well-known effect on protein structural dynamics at the so-called glass transition at 200 K (Karplus et al. 2000; Ringe and Petsko 2003). These, however, are totally different themes to what we emphasise here.

**Fig. 1:**
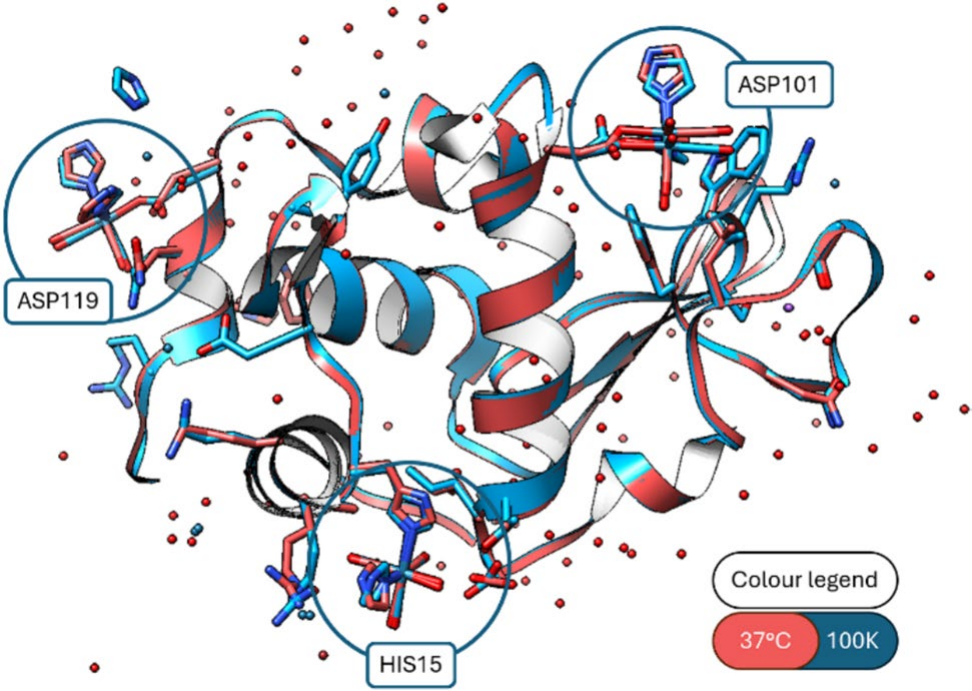
A ribbon diagram overlay of the 37 °C and 100 K protein structures. The three covalent binding sites at His15, Asp101 and Asp119 (blue model = 100 K, PDB code 8QCU; red model = 37 °C, 9GHX) are emphasised by blue circles. Figure is reproduced with permission of RSC Chem. Comm. (Jacobs et al. 2024)

Our interest in body temperature protein–ligand interactions stems from our research towards radiopharmaceutical drug development utilising the Group 7 transition metal chemical congener pair of rhenium for therapeutic application and its radioactive counterpart, technetium-99m for diagnostic application (Jürgens et al. 2014; Frei et al. 2018; Brink et al. 2022; Alberto 2023). The feasibility of metal-bound body temperature protein crystallography was reported (Jacobs et al. 2024) and considered differences in structure (e.g. number of bound waters) that were due to resolution variations (1.15 Å at 100 K versus 2.19 Å at 310 K) versus the conservation of the covalently bound *fac*-[Re(CO)₃-imidazole] complex moieties at 37 °C. The retention of the binding of an organometallic rhenium complex, a potential theranostic agent containing two metal centres (^186/188^Re + ^99m^Tc), to a protein at physiological body temperatures is an important factor for kinetically dominated transition metal coordination chemistry (Fig. 1).

It is noted that the pharmaceutical regulatory authorities’ requirement for medicines prescribed by medical practitioners in tablet form should be uniquely identified by powder X-ray diffraction (FDA 2007) as well as their precise chemical composition. Perhaps a future consideration, for compounds illustrating diverse reactivity, would be to characterise the medicinal compound at body temperature. This may provide key information for metallo-prodrug systems as the rate constant of transition metal complexes is known to double for each 10 °C increase in temperature in chemical reaction kinetic studies (Moore and Pearson 1981; Wilkins 2002) as illustrated in the ligand substitution kinetics of rhenium (Brink et al. 2013; Jacobs et al. 2021).

The above examples indicate the distinction which should be carefully made for structural details that change at the various temperatures both for experimental conditions (i.e. soaking, co-crystallisation, incubation, etc.) and experimental analysis (using XRD, MS, NMR, etc.). Importantly, during analysis, it should be noted which aspects become visible at the cryogenic temperatures that were not visible before at room temperature, likely due to improved diffraction data (at higher resolution) or thermodynamic and kinetic parameters which are temperature dependent. Halle (2004) emphasised that low occupancy ligand binding at 100 K was vulnerable to being an artefact of that low temperature, and it is this point that is especially important in new drug discovery. Indeed, this is not to say that 100 K cryo-macromolecular crystallography ought to be replaced. Structure-based drug discovery at 100 K is efficiently implemented at many MX beamlines worldwide and has led to notable discoveries, but it may be prudent to check favoured ligand binding events, particularly for ligand ‘drug-lead’ interactions at cryo-temperatures and at 37 °C. This, of course, is not guaranteed to be experimentally easy to execute.

In a recent review, the practical aspects of preparing, acquiring and analysing X-ray crystallography data at temperatures between 273 and 350 K were discussed and included mention of the limited number of structures reported at the higher temperatures, constrained by radiation damage (Fischer 2021). Indeed, the advantage of cryogenic X-ray crystallography has many benefits such as easy handling and transport of crystals to synchrotrons for remote data collection as well as long-term storage and reducing dehydration. A marked advantage of cryo-temperature data collections is to enable reduced X-ray radiation damage (Garman 2003; Weik and Colletier 2010; Garman and Weik 2023; Shelley and Garman 2024). A perspective by Hough et al. (2023) too described the recent developments on these topics.

To extend the possibility of temperature considerations further, it would be of interest for the study of living organisms that can thrive under hostile and harsh conditions. Could the atomic structure of thermophilic proteins and extremophilic proteins be studied at these organisms’ preferred ‘living’ temperatures, i.e. between 60 and 80 °C and > 80 °C? Radiation damage by X-rays will presumably become the limiting factor for protein X-ray crystallography, provided those protein crystals to be studied are mechanically stable, but here the advantages of the biophysical methodologies range would occur with the use of neutrons for the crystallography (Zaccai 2000; Zaccai and Coquelle 2020; Chen et al. 2012; Blakeleyet al. 2004) or by solution NMR. Additionally, the use of modelling tools, such as GeoPoc, may assist the prediction for the protein optimal temperature, pH, and salt concentration needed to enhance protein thermostability (Zhu et al. 2024). A structural comparison for mesophilic, moderately thermophilic and extremely thermophilic protein subunits has been made (Szilágyi and Závodszky 2000); however, the focus was on PDB structures with good data quality and specified the optimum growth temperature of the organisms. The review did not primarily address the analysis of data collected at different temperatures.

It should be noted that the majority of protein structures are crystallised either at low (~ 4 °C) or room temperature (18–22 °C) to promote nucleation, and to limit degradation problems as well as bacterial growth (Chayen 2004). Leading examples of protein–ligand interactions studied at physiological temperatures are described by Merlino et al. (Ferraro et al. 2016; Tito et al. 2025), whereby metalloproteins are being grown and analysed (by X-ray diffraction, electron paramagnetic resonance, UV–Vis spectroscopy, etc.) at temperatures ranging from 20 to 55 °C and notably at 37 °C.

The potential of crystallographic data collected at physiological body temperature could arguably be significantly expanded. A search of the RCSB PDB (https://www.rcsb.org) reveals interesting temperature trends. The search criteria were limited to X-ray diffraction experiments, searching specifically for crystallographic sample temperature during data collection at 100 K, 25 °C, 37 °C and any above 37 °C. We have excluded structures obtained from cryoEM (with the exception of the descriptive example in the ‘Electron cryo-microscopy (cryoEM)’ section), neutron diffraction or powder X-ray diffraction (PXRD) but have considered NMR analyses above 100 K. X-ray crystallographic structures at other temperatures, i.e. 0, 4 or 16 °C, common during the pre-1990’s, i.e. before the 3rd-generation synchrotron facilities commenced their operations, were also excluded. Our analysis also does not include earlier PDB X-ray crystallographic structures where the metadata of sample collection temperature was not mandatory at the time of PDB submission or where it is not listed. Also, the temperature of crystallisation by depositors is not always considered as a factor, only the temperature at which the crystallographic data collections were conducted. The data statistics revealed are shown in SI Fig. 1 (numerical values indicated in SI Table 1 and extended bin analysis is found in SI Fig. 2), which displays the trend of PDB entries versus the temperatures shown per year.

**Fig. 2:**
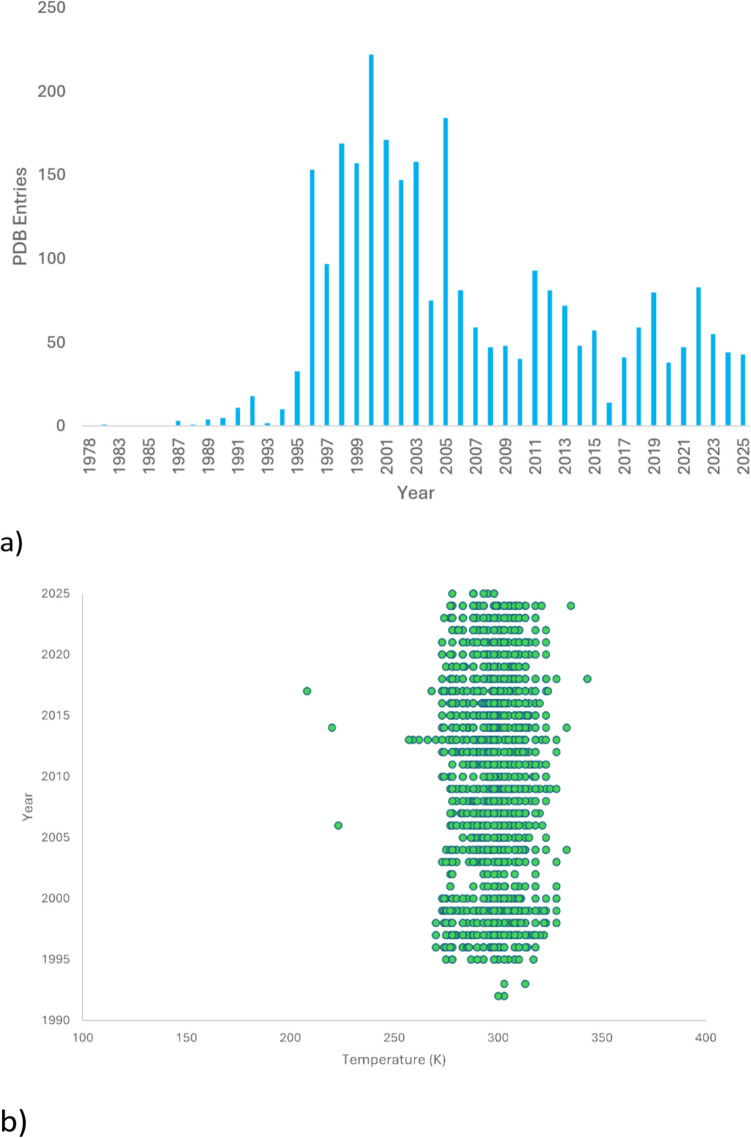
**a** X-ray crystallographic PDB entries for room temperature (25 °C) grouped per year. **b** Solution state NMR PDB entries indicated for all sample temperature analyses above 100 K per year

**Fig. 3:**
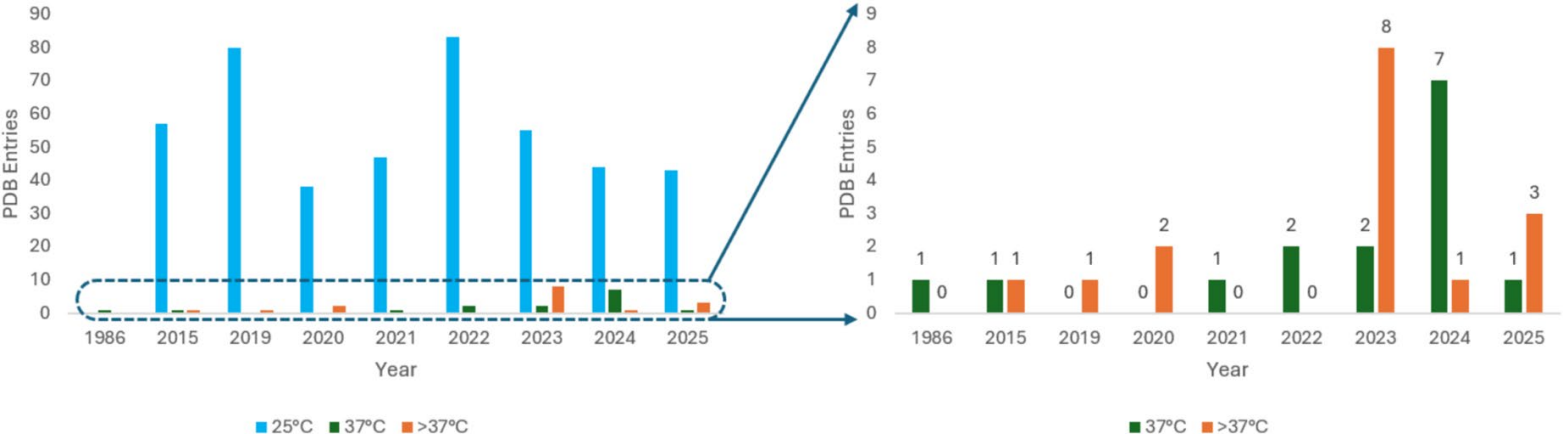
X-ray crystallographic PDB entries analysed at room temperature (25°C) versus those sampled obtained at 37 °C and >37°C, grouped per year (left-hand image). The right-hand image shows the actual numbers of data collections collected at these higher temperatures

Overall, the 100 K X-ray diffraction data clearly dwarf the data measured at the other temperatures. The relative contribution of earlier data sets at various temperatures were fairly equivalent, but at the turn of the twenty-first century, the number of the 100 K data collections increased dramatically (SI Fig. 1). During the past 10 years (2014–2024), there is an average PDB entry number of ca. 7000 entries per year. For data collected at 25 °C, there is an increasing trend up to the year 2000, and then the number of entries declined gradually in conjunction with the marked increase in cryo-data sets (Fig. 2a and SI Fig. 1); however, it is still significantly more in number than those obtained at ≥ 37 °C (Fig. 3). This contrasts markedly with NMR PDB entries, which are clustered around 300 K (Fig. 2b) over the past three decades.

**Fig. 4:**
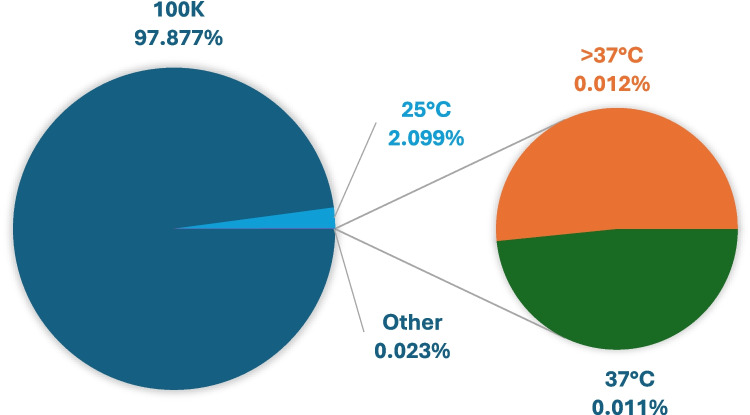
Relative contribution of X-ray crystallographic PDB entries obtained at 100 K versus those at room (25°C) and high temperatures (37°C and >37°C) as seen in the PDB

In summary, approximately 97.877% of available X-ray diffraction data published on RCSB PDB is collected at cryo-temperatures versus 2.099% at 25 °C and 0.023% collected at 37 °C and above. At the present date of publication, there are 15 PDB released entries at 37 °C and 16 PDB entries above 37 °C. Figure 4 highlights the vast number of cryogenic macromolecular structures contributing to our understanding of atomic models.

To sum up this section of our review, conducting crystallography checks at physiologically relevant temperatures, certainly body temperature 37 °C, is challenging yet feasible and could be a routine objective where physiologically relevant structural results are needed. Coming back to the question of the radiation damage by X-rays being increased considerably at 37 °C, the use of the neutron, X-ray laser and extremely bright synchrotron X-ray facilities is encouraged to add a body temperature setting to their sample temperature environment for diffraction data collection. Experimental setups, including the control of relative atmospheric humidity of the crystal during data collection, will certainly assist in obtaining adequate data, as seen for the study of conformational ensemble changes of SARS-CoV-2 main protease over a range of temperatures 100 K to 310 K (Ebrahim et al. 2022) where little to no evidence of radiation damage was observed even at the higher temperatures, albeit probably exceptional sample stability. Additional robust and applicable approaches to obtain high temperature crystallographic data are described by Doukov et al. (2020) which are complementary to room temperature serial femtosecond crystallography (SFX) data collections at X-ray free-electron lasers (XFELs).

## Other protein structure analytical methods

### Mass spectrometry

**Fig. 5:**
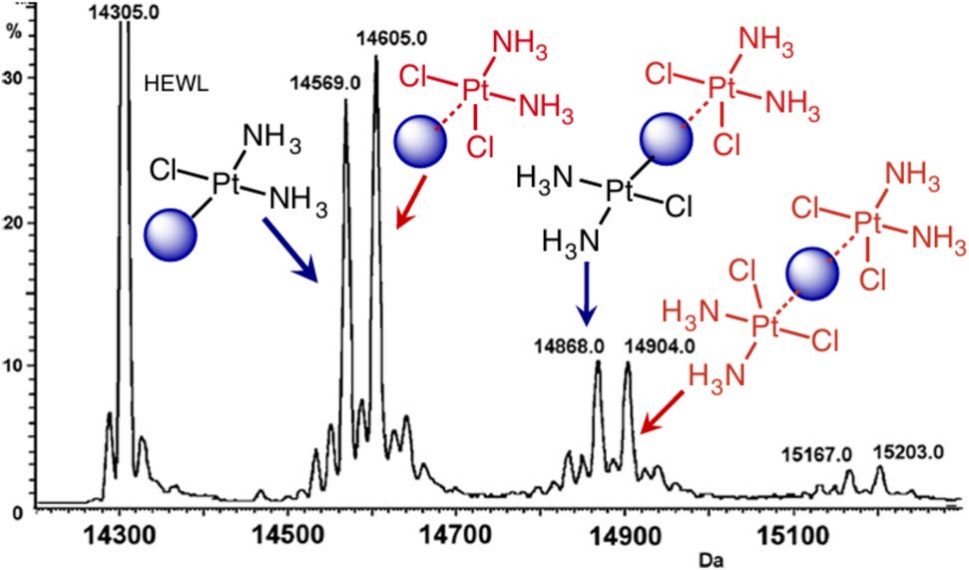
Deconvoluted electrospray ionisation mass spectrometry (ESI MS) spectrum of adducts formed between hen egg white lysozyme (HEWL) and cisplatin, after 48 h of incubation at 37 °C. The initial platinum/protein stoichiometry of each sample was 3:1. The blue sphere is a simplified representation of the globular protein in each sketch to which the platinum complexes are bound. The figure is reproduced with the permission of the author and Elsevier, (Casini et al. 2008)

In the absence of adequate crystals being available for structure elucidation, there are other biophysical methods that have followed the protocol of body temperature measurements which may be more appropriate for a particular study. A brief overview of methods which can provide molecular interactions and dynamic data at 37 °C is listed with examples below. An overview of mass spectrometry methods for measuring protein stability describes the effect of temperature measurement on interactions (Daneshfar et al. 2004; Tamara et al. 2022; Vallejo et al. 2022). Practical factors (including temperature settings) should be considered when analysing non-covalent interactions between peptides and metal ions or protein-small molecule interactions using electrospray ionisation-mass spectrometry (Carlton and Schug 2011; Lin and Gross 2022; Bennett et al. 2022). In the context of cancer treatment using platins, the use of mass spectrometry has been growing in its capabilities and can be applied to other therapeutic metallodrugs which can undergo extensive speciation in physiological environments (Hartinger et al. 2013; Wenzel and Casini 2017). The advantages and limitations of the method for analysing protein-metal interactions are mentioned by those authors. An example of anti-cancer covalent bound protein complexes analysed by mass as a complement to X-ray crystallography is provided by authors Casini et al. (2008), which utilise deconvoluted ESI–MS to analyse the metal-protein adducts formed after incubation at 37 °C (Fig. 5).

More generally Casini et al. (2008) also remarked:

‘*Real cellular systems are of course extremely more complicated as they may contain thousands of different proteins, at highly variable concentrations, and also a conspicuous number of low molecular weight components, most of them in relatively high concentration. In addition, these various components are highly organised and compartmentalised within the various intracellular structures thus offering a peculiar spatial distribution inside cells. It is obvious that studying the interaction of metallodrugs with proteins inside cells represents today a formidable challenge for researchers’.* Quoted with permission from Elsevier and the author.

The advantages of mass spectrometry for the analysis of metal prodrugs, with its varied and organometallic speciation, is the versatility of the technique, the relatively fast analysis of the samples and importantly the presence of the analyte in physiologically relevant conditions. The use of the two independent approaches (X-ray crystallography and mass spectrometry) provides complementary and valuable information on the total system. The one illustrates at atomic level the stationary state captured within a crystal, whilst mass spectrometry can provide information on the kinetic time changes of metal-protein interactions.

### Nuclear magnetic resonance (NMR)

**Fig. 6:**
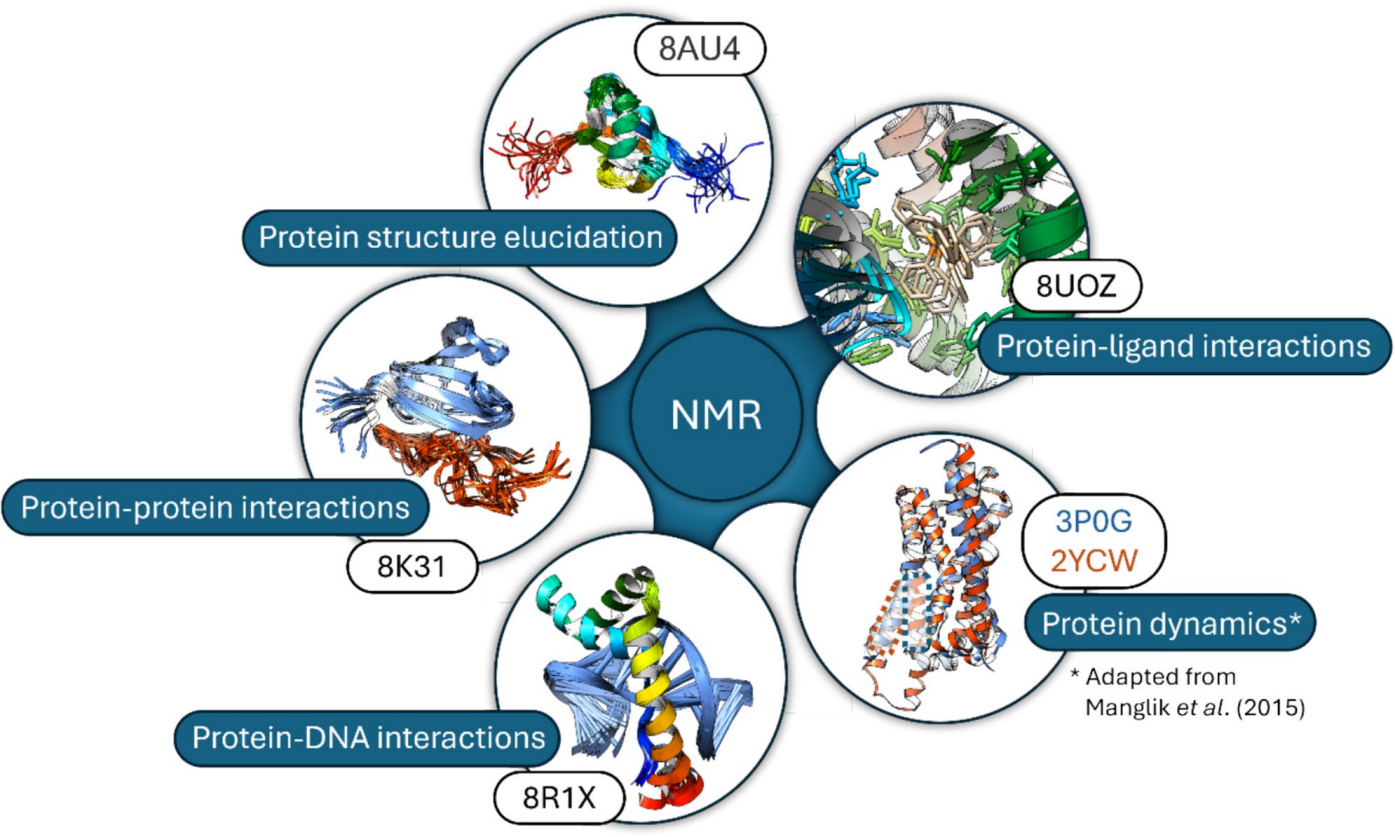
Various protein interaction structural studies at physiological conditions can be conducted by NMR as illustrated by representative examples listed. The PDB entry code is given in each case

The nuclear magnetic resonance of a protein in solution clearly has a major advantage over crystallography in that the NMR is a non-damaging probe and the mechanical stability or environment of a protein crystal does not feature as a necessary consideration. NMR is capable of detecting changes in the local electronic environment provoked by binding events, elucidating the regions of a protein involved in a binding interface. It is particularly advantageous, due to its sensitivity for weakly bound target-ligand complexes (Kerber et al. 2023). It therefore allows for atomic-resolution information on the structure as well as the possible dynamics and the thermodynamics of the protein–protein or protein–ligand interactions whilst being near physiological conditions (Purslow et al. 2020). Several NMR experimental methods to determine the atomic resolution of possible interactions can be conducted, such as chemical shift perturbation, solvent-paramagnetic relaxation enhancement and nuclear Overhauser effects. Additionally, protein–ligand interactions in both solution and solid state can be analysed (Watts 2005; Miao and Cross 2013). Unlike crystallography, NMR does not allow the solvent to be modelled in a simple way, and the method remains restricted to proteins of limited size (Kwan et al. 2011). A few selected examples of various protein NMR studies (below text and Fig. 6) illustrate the range of the research and analytical findings which can be obtained.

**Protein structure elucidation** was used to investigate the Ras isoforms for their prevalence as mutations in several cancers. The binding interface of KRas4B^G12V^:Rgl2^RA^ was investigated crystallographically, where Rgl2 is considered to play a critical role in Ras-induced tumour phenotypes (Vigil et al. 2010). The structure of free Rgl2^RA^ was then elucidated by NMR and compared to the crystallographic structure, which was found to be very similar (Tariq et al. 2024). Solution NMR over a 30–37 °C range was used to define the allosteric effects in Cyp33, an RNA binding protein, for ongoing efforts in understanding leukaemia, especially in infants for future disease treatment and/or prevention (Blatter et al. 2023).

**Protein-protein interactions** including intrinsically disordered proteins can be studied by NMR up to atomic resolutions as seen for WRKY33, a transcription factor in plants (PDB Code: 8K31; (Dong et al. 2024).

**Protein-DNA binding** Solution NMR studies are useful for determining the structures of protein systems where ligand binding is either strong or comparatively weak, the system being too small for electron microscopy or too flexible for crystallography (Sugiki et al. 2017). DNA binding with a high-mobility group box protein (HMG-D) was studied at room temperature (PDB Code: 8R1X) and atomic resolution interactions could be identified (Hill et al. 2024).

**Protein–ligand interactions** Membrane proteins can be difficult to work with crystallographically (Martin and Sawyer 2019). NMR techniques bypass the crystallisation step and allow for the study of such a protein provided that proper buffers and detergents are added to stabilise the protein (Rawlings 2016). Li and coworkers looked at the drug recognition mechanism of the multidrug efflux transporter EmrE, a membrane protein, using NMR and could identify which residues are interacting with the tetraphenylphosphonium ligand throughout the mechanism (Li et al. 2024). Notably, in the scope of this review, this study was conducted at 37 °C, a temperature easily achievable for modern NMR instruments.

**Protein dynamics Dynamic** systems in solution can be investigated; one example utilised ^19^F NMR studying the structural dynamics of the β_2_-adrenergic receptor with a focus on the cytoplasmic domain of the protein (Manglik et al. 2015). Depicted in the figure in the protein dynamics inset are the two positions of the protein as seen in their respective crystal structures of the active (PDB Code: 3P0G (Rasmussen et al. 2011)) and inactive conformations (PDB Code: 2YCW (Moukhametzianov et al. 2011).

The folding and unfolding rates of two engineered apocytochrome *b*_562_ variants were studied with ^15^N relaxation dispersion NMR spectroscopy over a range of temperatures of 37.5 − 47.5 °C (Choy et al. 2005). Indeed, protein folding and unfolding as a function of temperature using NMR is an extensive research field, as described by Fersht (see chapters 18 and 19 in Fersht (1999)). Additionally, a ^13^Cα-^13^CO correlation NMR experiment was developed to monitor site-specific phosphorylation at high pH and temperature (283–310 K), conditions which are necessary for kinase activity, and thus obtained residue-specific information on disordered proteins (Alik et al. 2020). NMR data have also been used to calculate hydrophobicity propensities for all amino acids at five different temperature ranges (spanning 265–340 K), an approach which showed that the hydrophobic effect becomes weaker at lower temperatures, as expected from theoretical predictions (van Dijk et al. 2015).

In conclusion, NMR can support the emergence of classical structural biology research with cellular biology research to understand the great complexity of the living cell as highlighted by the mass spectrometry research reported by Casini et al. (2008). ‘In-cell NMR’ is an approach which can be applied to obtain structural and functional information on biological macromolecules inside intact, living cells. Hence, this approach allows for true cellular structural biology to allow for macromolecules to be characterised directly in their native environment (Luchinat and Banci 2017).

### X-ray free-electron lasers (XFELs)

X-ray free-electron lasers (XFELs) experiments allow for crystallographic and spectroscopic structure analysis as well as imaging, thus spanning a wide range of length scales within timescales from femtoseconds to seconds. For the structure investigation of submicrometre sized crystals, which are typically too small for conventional crystallography, it is a breakthrough complementary to electron crystallography. Most importantly, in the context of analysing ‘living systems’, it allows for time-resolved diffraction at room or body temperature whilst avoiding many of the effects of X-ray irradiation damage. The ‘diffract-then-destroy’ method can, for example, use X-ray pulses of 10–40 fs in duration to produce a diffraction pattern before the sample is destroyed. A continuous stream of new crystals runs before the pulsed beam, and the diffraction images are merged at a later date (thumbnail contents image, figure inset). The need to cool samples to minimise radiation damage is thereby no longer a priority, and therefore this technique permits atomic resolution imaging of molecular processes on the 100 fs timescale under near-physiological conditions and in a temperature-controlled environment close to ideal protein operational conditions. This allows for protein structural dynamics to be investigated at room temperature. Whilst 37 °C studies from XFEL have not started in earnest, we foresee substantive analysis for the future. A series of excellent reviews and developments on XFELs for structure analysis have been published recently (Spence 2017; Lee et al. 2018; Caramello and Royant 2024).

### X-ray photon correlation spectroscopy (XPCS)

A new and developing method is that of X-ray photon correlation spectroscopy (XPCS) (Perakis and Gutt 2020). XPCS is a method to measure dynamics on time scales of 1 s–10^–12^ s in condensed matter systems by correlating time-resolved coherent diffraction patterns to monitor nanoscale fluctuations such as in crowded protein solutions of a biological cell. Coherent X-rays, which are monochromatic, are required as provided by an X-ray laser. The X-ray intensity is attenuated to avoid beam-induced effects. The scattering intensity as a function of time is recorded with a pixelated 2D array detector which fluctuates due to the changes of the speckle pattern. The measurement of this fluctuating speckle pattern must balance the reduction of the random errors of the photon counting fluctuations and yet restrict the systematic error that would arise from radiation damage to the sample. This new method aims to provide time-resolved imaging of functionally relevant living processes*.* XPCS lends itself to making measurements at body temperature. This new method is under active development at the Stanford Linac coherent light source (Alonso-Mori et al. 2015), EuroXFEL (Madsen et al. 2021) and at Spring8/SACLA (Tanaka et al. 2024). Applications to a wider range of examples in biophysics will follow these early applications given the enthusiastic activity of the facilities and their user groups.

### Nano differential scanning fluorimetry (nanoDSF)

Nano differential scanning fluorimetry (nanoDSF) is a high-throughput protein stability screening technique that simultaneously monitors protein unfolding and aggregation properties. It is a type of differential scanning fluorimetry method, conducted in the absence of external dyes, that monitors the change of intrinsic fluorescence from tryptophan found in protein as a function of temperature, time or denaturant concentration (Kim et al. 2021; Lisina et al. 2023). A methodology description is provided that describes means to determine the ligand binding through temperatures ranging from 20 to 95 °C (Ahmad et al. 2021).

### Electron cryo-microscopy (cryoEM)

The use of cryoEM to study a sample at 37 °C then flash frozen is especially noteworthy (Hu et al. 2024). Conformational changes dependent on a temperature-sensitive Ca^2+^-activated ion channel affected the structure and function of TRPM4. There were distinct properties of TRPM4 at physiological temperatures (37 °C) versus room temperature that prompted investigation of the molecular mechanism of TRPM4 under physiological temperatures. The study emphasises the value of studying macromolecules at physiological temperatures.

## Overall summary

To mention just one example, the International Union of Pure and Applied Biophysics Congresses bring together a host of different characterisation methodologies and results appropriate for the atomic and molecular level of detail through to the cellular and whole organism scale. These meetings of biophysics experts have long thrown down a gauntlet to such as X-ray protein crystallographers to determine body temperature structural results if the crystal growth or stability allows for such experiments. This is an especially pressing need in our view with respect to the field of theranostics, which we are engaged in. Our review article sets the body temperature protein crystallography research and development in the context of other molecular biophysics methods such as NMR, mass spectrometry and cryoEM. We document that other fields such as mass spectrometry and NMR have established precedents and experimental limitations better suitable for variable temperature analysis. Contributions combining methodologies that allow for best resolution experimental data plus analysis closer to physiological ‘life’ conditions show that progress is possible. The combinatory practice of methods brings together more evidence and perspectives, as exemplified by the statement of Astbury (1948), ‘We need much more cooperation, much more fraternization, much more encouragement of one another, much more sharing of knowledge and much more asking of help’ and supports the improvement of the pharmaceutical regulation of new compounds for improved patient treatment.

## Supplementary Information

Below is the link to the electronic supplementary material.

ESM 1 (PDF 219 KB)

## Data Availability

No datasets were generated or analysed during the current study.
