# Supplementary material for: Macromolecular crystallography for mammalian body temperature in support of molecular biophysics methods
Source: Biophys Rev. 2025 Aug 1;17(4):947–57. doi: 10.1007/s12551-025-01328-4 (PMC12686291; doi:10.1007/s12551-025-01328-4)
Supplement: Supplementary file 1 — (PDF 219 KB) [file 12551_2025_1328_MOESM1_ESM.pdf]

Macromolecular crystallography for mammalian body temperature structures in support of molecular biophysics methods

Alice Brink[a], John R Helliwell[b] and Francois Jacobs[a]

[a] Chemistry Department, University of the Free State, Nelson Mandela Drive, Bloemfontein, South Africa.

[a] Department of Chemistry, University of Manchester, Oxford Road, Manchester, UK

Corresponding authors: brinka@ufs.ac.za and john.helliwell@manchester.ac.uk

Supporting Information

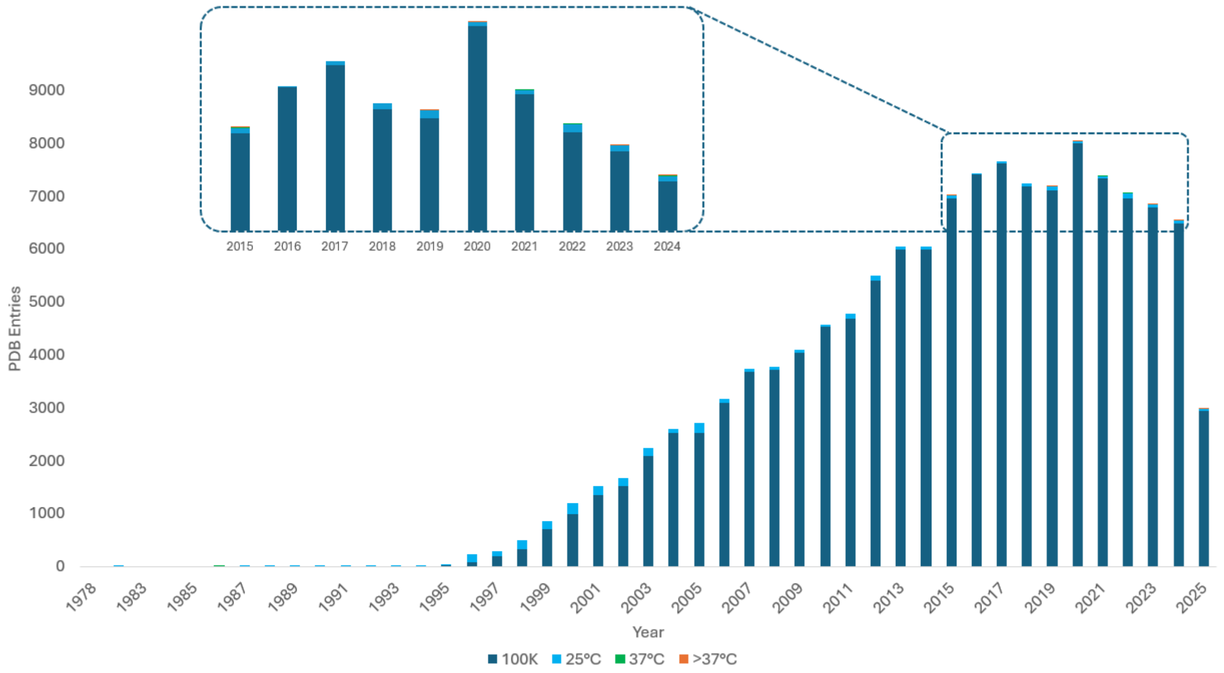

SI Fig.1. X-ray crystallographic structures available from the PDB obtained at low (100 K) and high (25°C, 37°C and >37°C) sample analysis temperatures plotted per year. The inset shows an enlarged section for the years 2015-2024 to clearly highlight the notable disparity between the high (≥37°C) and low temperature (100K ≤ 25°C) crystal data sets.

SI Table.1. The X-ray crystallographic structures at low (100 K) and high (25°C, 37°C and >37°C) temperatures tabulated per year as found on the PDB.

	Number of PDB entries			
**Year**	**100K**	**25°C**	**37°C**	**>37°C**
**1978**	0	0	0	0
**1982**	0	1	0	0
**1983**	0	0	0	0
**1984**	0	0	0	0
**1985**	0	0	0	0
**1986**	0	0	1	0
**1987**	0	3	0	0
**1988**	0	1	0	0
**1989**	1	4	0	0
**1990**	1	5	0	0
**1991**	0	11	0	0
**1992**	3	18	0	0
**1993**	1	2	0	0
**1994**	4	10	0	0
**1995**	18	33	0	0
**1996**	75	153	0	0
**1997**	194	97	0	0
**1998**	336	169	0	0
**1999**	702	157	0	0
**2000**	984	222	0	0
**2001**	1343	171	0	0
**2002**	1524	147	0	0
**2003**	2083	158	0	0
**2004**	2519	75	0	0
**2005**	2534	184	0	0
**2006**	3089	81	0	0
**2007**	3688	59	0	0
**2008**	3724	47	0	0
**2009**	4049	48	0	0
**2010**	4525	40	0	0
**2011**	4692	93	0	0
**2012**	5413	81	0	0
**2013**	5984	72	0	0
**2014**	5998	48	0	0
**2015**	6952	57	1	1
**2016**	7407	14	0	0
**2017**	7625	41	0	0
**2018**	7190	59	0	0

**2019**	7104	80	0	1
**2020**	8003	38	0	2
**2021**	7334	47	1	0
**2022**	6961	83	2	0
**2023**	6783	55	2	8
**2024**	6489	44	7	1
**2025**	2942	43	1	3
**Total**	128274	2751	15	16

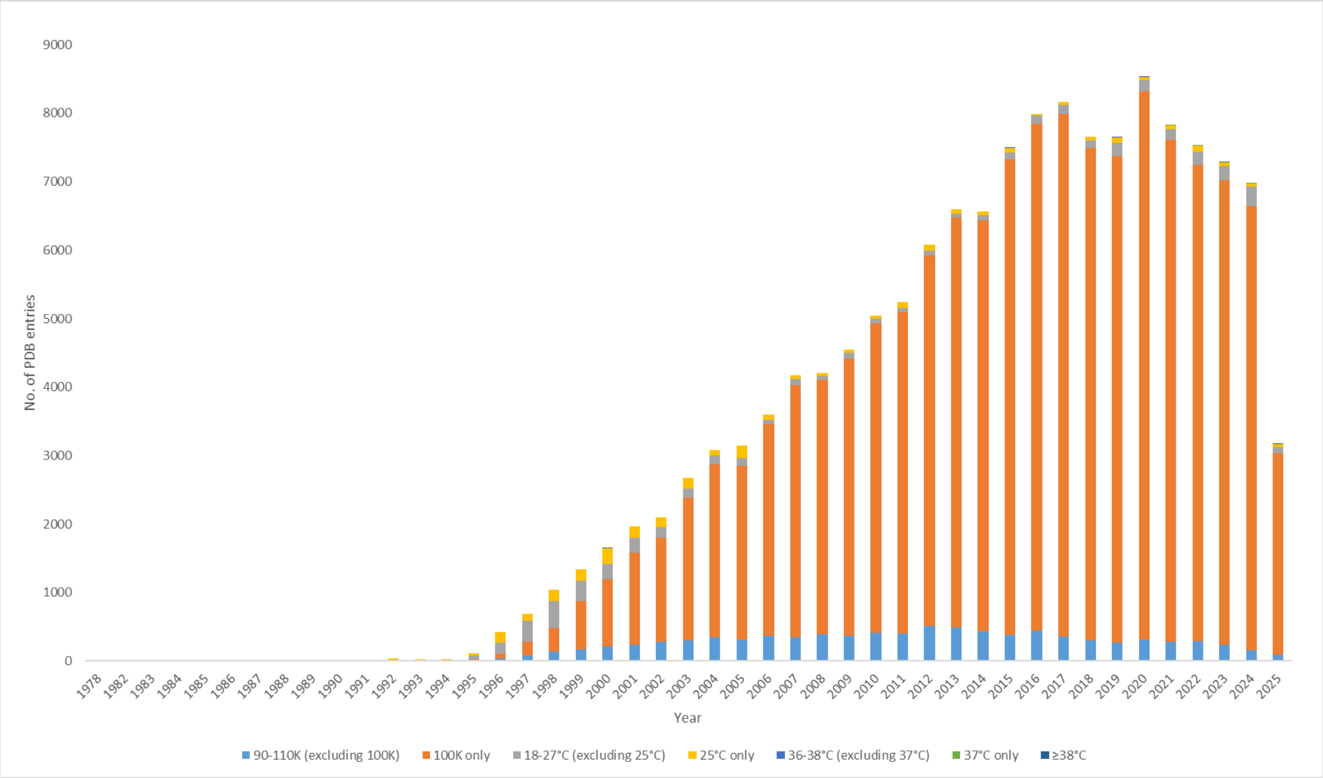

SI Fig.2. Extended data bin range of X-ray crystallographic structures available from the PDB indicating sample analysis temperature versus year published. The data is indicated within bin ranges of 90-110 K (bottom light blue, excluding 100 K); 100 K only (middle orange); 18-27 °C (top grey, excluding 25°C); 36-38°C (top blue, excluding 37°C); 37°C only (top green) and >38°C (top navy-blue). Data collections sampled at low temperature 90-110K (bottom light blue) + 100K (middle orange) clearly indicate the marked number of low temperature data versus the barely visible, high temperature ranges (18-38°C) indicated at the top of the histograms.

SI Table.2. Extended data bin range of X-ray crystallographic structures available from the PDB indicating sample analysis temperature versus year published (data visually indicated in Fig SI 2).

	**Number of PDB entries**						
**Year**	**90-110K (excluding 100K)**	**100K only**	**18-27°C (excluding 25°C)**	**25°C only**	**36-38°C (excluding 37°C)**	**37°C only**	**≥38°C**
**1978**	0	0	0	0	0	0	0
**1982**	0	0	1	1	0	0	0
**1983**	0	0	0	0	0	0	0
**1984**	0	0	0	0	0	0	0
**1985**	0	0	0	0	0	0	0
**1986**	0	0	0	0	0	1	0
**1987**	0	0	3	3	0	0	0
**1988**	0	0	1	1	0	0	0
**1989**	0	1	4	4	0	0	0
**1990**	0	1	2	5	0	0	0
**1991**	1	0	2	11	0	0	0
**1992**	0	3	12	18	0	0	0
**1993**	1	1	9	2	0	0	0
**1994**	2	4	13	10	0	0	0
**1995**	7	18	54	33	0	0	0
**1996**	32	75	166	153	0	0	0
**1997**	85	194	308	97	0	0	0
**1998**	138	336	398	169	0	0	0
**1999**	172	702	302	157	0	0	0
**2000**	213	984	223	222	1	0	0
**2001**	235	1343	218	171	0	0	0
**2002**	275	1524	154	147	0	0	0
**2003**	302	2083	131	158	0	0	0
**2004**	348	2519	136	75	0	0	0
**2005**	317	2534	111	184	0	0	0
**2006**	364	3089	69	81	0	0	0
**2007**	345	3688	79	59	0	0	0
**2008**	386	3724	46	47	0	0	0
**2009**	369	4049	82	48	0	0	0
**2010**	412	4525	65	40	0	0	0
**2011**	405	4692	52	93	0	0	0
**2012**	509	5413	73	81	0	0	0
**2013**	488	5984	58	72	0	0	0
**2014**	438	5998	77	48	0	0	0
**2015**	376	6952	102	57	1	1	1

**2016**	440	7407	123	14	0	0	0
**2017**	361	7625	139	41	0	0	0
**2018**	306	7190	102	59	0	0	0
**2019**	269	7104	191	80	0	0	1
**2020**	314	8003	173	38	0	0	2
**2021**	278	7334	160	47	0	1	0
**2022**	290	6961	190	83	0	2	0
**2023**	236	6783	211	55	0	2	8
**2024**	157	6489	279	44	0	7	0
**2025**	94	2942	91	43	0	1	3
**Total**	8965	128274	4610	2751	2	15	15
